# Matrix-metalloproteinase-9 is cleaved and activated by Cathepsin K

**DOI:** 10.1186/s13104-015-1284-8

**Published:** 2015-07-29

**Authors:** Jon Christensen, V Prasad Shastri

**Affiliations:** Institute for Macromolecular Chemistry, University of Freiburg, Hermann-Staudinger-Haus Stefan-Meier-Straße 31, 79104 Freiburg, Germany; BIOSS-Centre for Biological Signalling Studies, University of Freiburg, Schänzlestraße 18, 79104 Freiburg, Germany

**Keywords:** Enzyme activation, MMP-9 activation, CTSK, Bone resorption, Cancer therapy

## Abstract

**Background:**

Matrix-metalloproteinases 9 (MMP-9) belongs to the class of matrix metalloproteinases whose main function is to degrade and remodel the extracellular matrix (ECM). MMP-9 has been shown to be an integral part of many diseases where modulation of the ECM is a key step such as cancer, osteoporosis and fibrosis. MMP-9 is secreted as a latent pro-enzyme that requires activation in the extracellular space. Therefore, identifying physiological and molecular contexts, which can activate MMP-9 is important.

**Results:**

Acidification of osteoclast-conditioned media to pH 5 resulted in a fragment with a size corresponding to active MMP-9. Also, treatment of recombinant proMMP-9 with recombinant cathepsin K (CTSK) at pH 5 yielded a fragment that corresponded to the molecular weight of active MMP-9, and showed MMP-9 activity. This activation was abrogated in the presence of CTSK inhibitor indicating that CTSK was responsible for the activation of pro-MMP-9. Knocking down CTSK in MDA-MB-231 cells also diminished MMP-9 activity compared to wild type control.

**Conclusions:**

Here we provide the first evidence that CTSK can cleave and activate MMP-9 in acidic environments such as seen in tumors and during bone resorption. This finding provides a key link between CTSK expression in tumors and bone and ECM remodeling, through MMP-9 activation. This novel mechanism to activate MMP-9 through extracellular physiological changes elucidated in this study reveals a protease-signaling network involving CTSK and MMP-9 and provides the impetus to explore ECM proteases as physiological markers and pharmacological targets.

**Electronic supplementary material:**

The online version of this article (doi:10.1186/s13104-015-1284-8) contains supplementary material, which is available to authorized users.

## Background

Matrix metalloproteinase-9 (MMP-9) is a zinc-dependent endopeptidase that participates in a variety of physiological and biochemical processes. In tumor biology, MMP-9 plays an important role in tumor progression and it has been shown that MMP-9 increases angiogenesis [1], cancer cell migration [2] and metastasis [3]. In the tumor microenvironment infiltrating cells, such as bone marrow cells [1], tumor associated macrophages [4, 5] and neutrophils [6] have been shown to be a rich source of MMP-9 and tumor cells themselves express higher levels of MMP-9 compared to healthy tissue [7]. Also, it has been shown that cancer cells can induce expression of proteolytic enzymes such as MMP-2, MMP-9 and urokinase in adjacent fibroblasts through paracrine signalling [8–10]. MMP-9 has also been shown to be important for bone development and repair. MMP-9 knockout mice exhibit altered growth plate vascularization and ossification during development and delayed repair of experimental induced bone fractures [11, 12].

MMP-9 is secreted as an inactive enzyme (proMMP-9) with a molecular weight of 92 kDa. A cysteine in the N-terminal pro-domain binds to the zinc atom in the active site thus maintaining latency [13]. Activation of MMP-9 requires a disruption of the cysteine interaction with the zinc atom thus exposing the catalytic site [13]. The most studied mechanism of MMP-9 activation is enzyme proteolysis of the pro-domain. Activators of MMP-9 include MMP-2 [14, 15], MMP-3 [14, 16], MMP-7 [17], MMP-10 [18], MMP-13 [19], cathepsin G [20] and urokinase/plasmin [21, 22]. Among these MMP-3 is considered the most potent activator of MMP-9 [23]. Activation of pro-MMP-9 by the aforementioned activators results in active MMP-9, which has a molecular weight in the neighborhood of 82 kDa. Post-activation processing of MMP-9 can lead to the removal of the C-terminal hemopexin-like domain (65 kDa species) or a direct cleavage of the active site leading to an inactive species (50–60 kDa) [24].

Lysosomal cysteine cathepsin (LCC) is a subgroup of the family of cathepsin proteases that requires an acidic environment for optimal activation, and is found mainly in the acidic lysosomes where they participate in protein turnover and degradation of internalized ECM [25]. CTSK is a member of the LCC sub group, but as opposed to other LCC, CTSK is mainly secreted into the extracellular space where it can degrade fibrillar collagen as found in bone and cartilage [26]. High CTSK expression has been found in osteoclasts (Ocs) and synovial fibroblasts, where it is responsible for the degradation of bone and articular cartilage [27, 28], respectively, and in cells associated with breast and prostate cancer metastatic lesions in bone [29–31]. Like CTSK, MMP-9 is also highly expressed in Ocs, and synovial fibroblasts where it contributes to degradation of the ECM [32, 33]. In bone metastasis MMP-9 is associated with loss of bone matrix and participates in catabolism of bone [34]. Due to the restricted expression profile of CTSK compared to other LCCs, and its overlap with the expression of MMP-9 we hypothesized that CTSK and MMP-9 might be involved in a protease-signaling network. We investigated this premise in this study and present evidence that CTSK can cleave and activate proMMP-9 under acidic conditions.

## Results and discussion

### Osteoclast-derived CTSK can process proMMP-9

Epidemiological studies show that incidence of bone metastasis is reduced in both early and late-stage breast cancer patients receiving bisphosphonate, suggesting a possible link between bone turnover and metastasis to the bone [35]. It is well established that tumor environments and breast and prostate cancer metastasis lesions in bone show high expression of CTSK. Elevated levels of MMP-9 have also been linked to breast cancer and tumor invasion [2]. Interestingly, CTSK expression has also been reported in the population of breast and prostate cancer cells that metastasize to bone [29–31]. However, why the acquisition of a CTSK expressing phenotype might be important for cancer cells still remains unanswered.

**Fig. 1:**
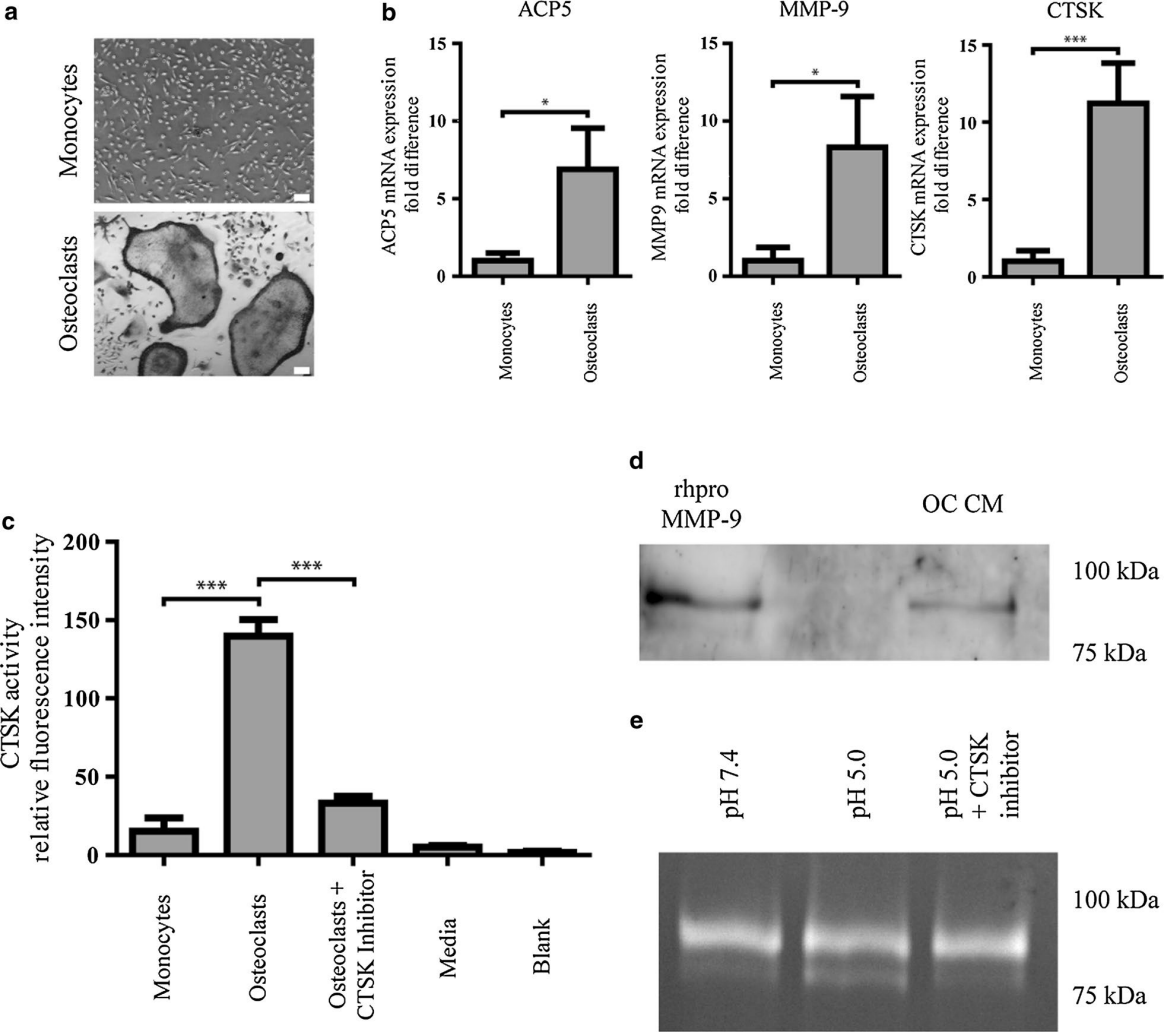
Characterization of human monocyte-derived osteoclasts secreted protease activity. **a** Bright field image of monocytes and differentiated osteoclasts stained for TRAP. *Scale bar* 10 µm. **b** qRT-PCR of freshly isolated CD14^+^ monocytes and differentiated osteoclasts. Values were first normalized to RRN18S and then to the gene expression levels in monocytes. Data is presented as the mean ± SD, n = 3. **c** CM from freshly isolated CD14^+^ monocytes and differentiated osteoclasts were analyzed for the presence of active CTSK. Data is presented as the mean ± SD, n = 3. **d** CM from Ocs were subjected to Western blot analysis and probed with an antibody against proMMP-9. 20 ng of rhproMMP-9 was used as a positive control. **e** Gelatin zymography of Oc CM. The pH of the CMs was maintained at 7.4 or lowered to 5.0 and incubated for 1 h at 37°C with or without a CTSK inhibitor. Afterwards, the samples were analyzed by gelatin zymography. *P < 0.05 and ***P < 0.005.

Since a key pathological trait of osteoporosis is increased osteoclastic activity we examined the expression levels of CTSK and MMP-9 in Ocs derived from human monocytes differentiated in the presence of RANKL and observed that in addition to morphological characteristics such as TRAP positive giant multinucleated cells, upregulation of CTSK and ACP5 mRNA (Fig. 1a, b) monocyte-derived Oc also express MMP-9 mRNA (Fig. 1b). Additionally, the Oc secretome showed secretion of active CTSK (Fig. 1c) and proMMP-9 (Fig. 1d) indicating that both CTSK and proMMP-9 are available in the extracellular milieu. We then posed the question, if proMMP-9 was a substrate for CTSK secreted by Ocs. To test this, conditioned media (CM) from Ocs was acidified to pH 5, with and without a CTSK inhibitor, and the fate of proMMP-9 was assessed by gelatin zymography. Bands in zymography corresponding to a calculated molecular weight of 99 and 87 kDa were observed, and this corresponded well with theoretical size of pro- and active- MMP-9 which is 92 and 82 kDa, respectively (Fig. 1e). Addition of CTSK inhibitor resulted in the loss of the zymography band at 87 KDa, indicating a potential role for CTSK in processing proMMP-9. Furthermore, the osteoclasts conditioned media was assayed for MMP-3 and MMP-3 was not detected (Additional file 1). This further strengthens the conclusion that CTSK is an activator of MMP-9.

### rhCTSK enzymatically activates rhproMMP-9

**Fig. 2:**
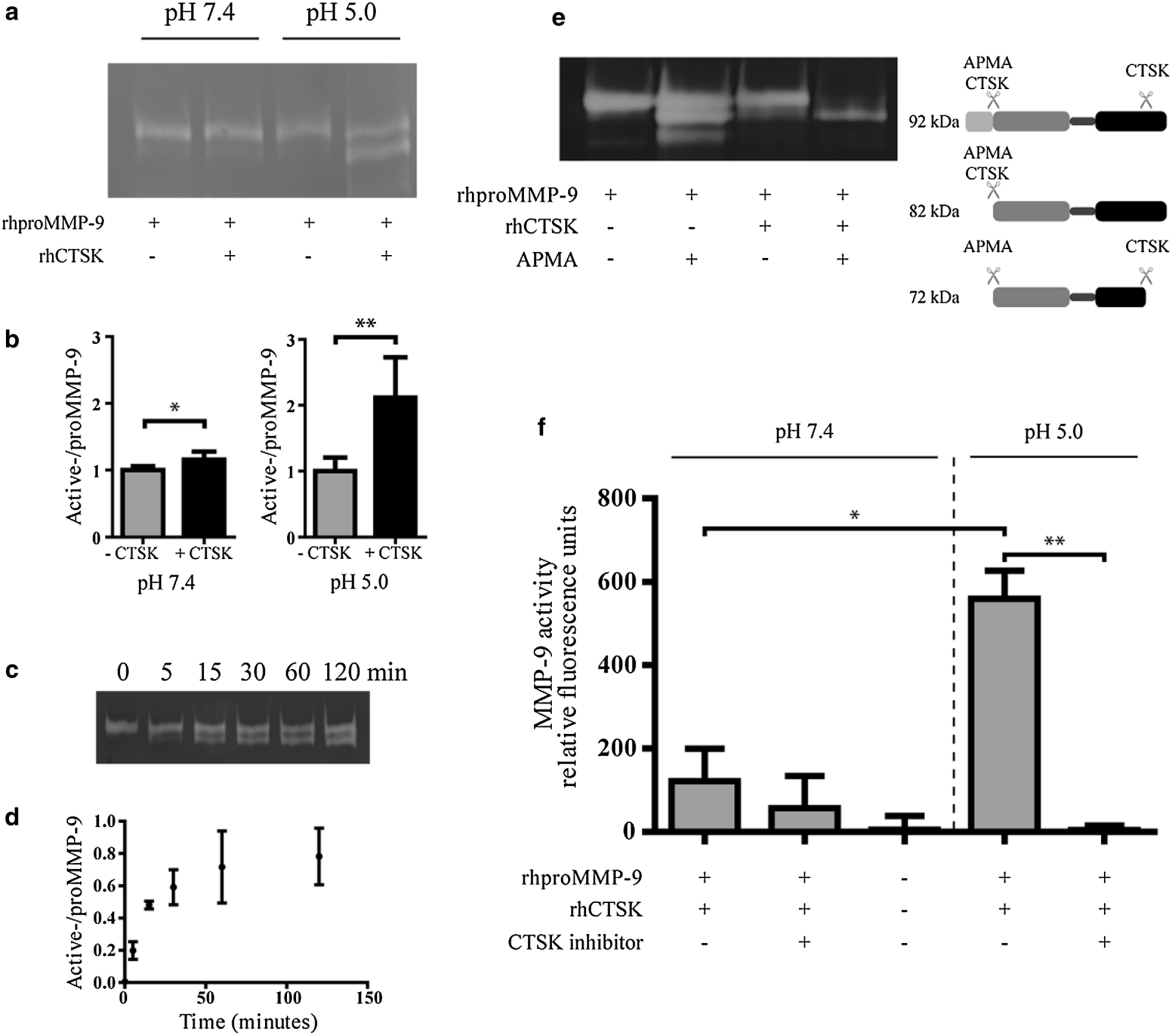
rhCTSK can cleave and activate rhproMMP-9 at acidic pH. **a** rhproMMP-9 (5.0 ng) was incubated with rhCTSK (0.5 ng) at pH 5.0 for the duration indicated, and then analyzed via gelatin zymography. **b** Quantification of active MMP-9 using the zymography data shown in **a**. Y-axis is the ratio between active- and proMMP-9, measured from the relative light intensity. Data is presented as mean ± SD, n = 3. **c** Zymograph of solutions of rhproMMP-9 (5.0 ng) incubated for 1 h, at 37°C with or without rhCTSK (0.5 ng) at pH 7.5 or pH 5.0. ** d** Quantification of zymography bands shown in **c**. Y-axis is the ratio between active- and proMMP-9, measured from the relative light intensity of the bands normalized to the control condition without rhCTSK. Data is presented as the mean ± SD, n = 8. **e** rhproMMP-9 (5.0 ng) was first incubated for 1 h at 37°C at pH 5.0 with or without rhCTSK (5.0 ng), and then pH was adjusted to 8.0 and incubated for an additional 2 h at 37°C with or without APMA (final concentration 1.5 mM), and then analyzed via gelatin zymography. **f** Quantification of MMP-9 activity using a fluorescently quenched substrate for MMP-9 (Mca-RPPGFSAFK(Dnp)). Data is presented as the mean ± SD, n = 3. *P < 0.05 and **P < 0.01.

To further confirm the above findings and to exclude the role of other secreted lysosomal cysteine proteases active under acidified conditions in the processing of proMMP-9, we used recombinant human proMMP-9 (rhproMMP-9) and CTSK (rhCTSK) and we observed that rhCTSK could also cleave rhproMMP-9 at pH 5 and yielded the same molecular weight fragments of MMP-9 as was observed with Oc CM (Fig. 2a, b). While, some cleavage of rhproMMP-9 by rhCTSK was also observed at physiological pH, it was evident that this process was most efficient at pH 5. A time course study revealed that the cleavage of rhproMMP-9 showed time dependence with an initial rapid increase in cleavage of rhproMMP-9 reaching a plateau after 1 h (Fig. 2c, d).

### rhCTSK cleavage of rhproMMP-9 results in enzymatically active rhMMP-9

Since the active site of MMP-9 is between the 107–444 AA residues, it is plausible that the enzymatic cleavage of rhproMMP-9 by rhCTSK at either or both the C- or N-terminus could give rise to similar molecular weight fragments that would still be detectable by zymography, as the catalytic domain would be retained in both instances. In order to gain insight into the site of action of rhCTSK within rhproMMP-9, we incubated rhproMMP-9 sequentially with rhCTSK followed by 4-aminophenylmercuric acetate (APMA) and determined the molecular weight of the fragments. Since APMA is known to promote the autocatalytic cleavage of rhproMMP-9 at the N-terminus, if rhCTSK cleaves at the N-terminal (pro-domain) [24], then a single 82 kDa fragment should be obtained. On the other hand if rhCTSK cleaves at the C-terminus (hemoplexin domain) then subsequent incubation with APMA would result in a further removal of a 10 kDa fragment, resulting in an additional 72 kDa fragment. However, treating rhproMMP-9 with rhCTSK and APMA gave rise to a rhMMP-9 fragment with a molecular weight of 82 kDa (Fig. 2e), providing evidence that in all likelihood APMA and CTSK act in the same vicinity within the prodomain of MMP-9. The presence of lower molecular weight fragment in the presence of APMA can be attributed to the autocatalytic cleavage of rhMMP-9 in presence of APMA, which is known [24].

To examine if the cleavage of rhproMMP-9 by rhCTSK yielded the enzymatically active rhMMP-9, we pre-treated rhproMMP-9 with rhCTSK and followed rhMMP-9 activity using a fluorescence assay. A marked increase in fluorescence intensity was observed in the conditions that involved acidification in presence of rhCTSK. To verify that this increase was indeed due to the action of rhCTSK on rhproMMP-9, the experiment was repeated in presence of a CTSK inhibitor. A 100-fold decrease in rhMMP-9 activity was observed suggesting that rhCTSK cleaves the rhproMMP-9 leading to its activation and provides circumstantial evidence for a protease-signaling network involving CTSK and pro-MMP-9 in vitro (Fig. 2f). This important finding need to be further investigated in vivo in animal models.

### Tumor derived CTSK activates proMMP-9

**Fig. 3:**
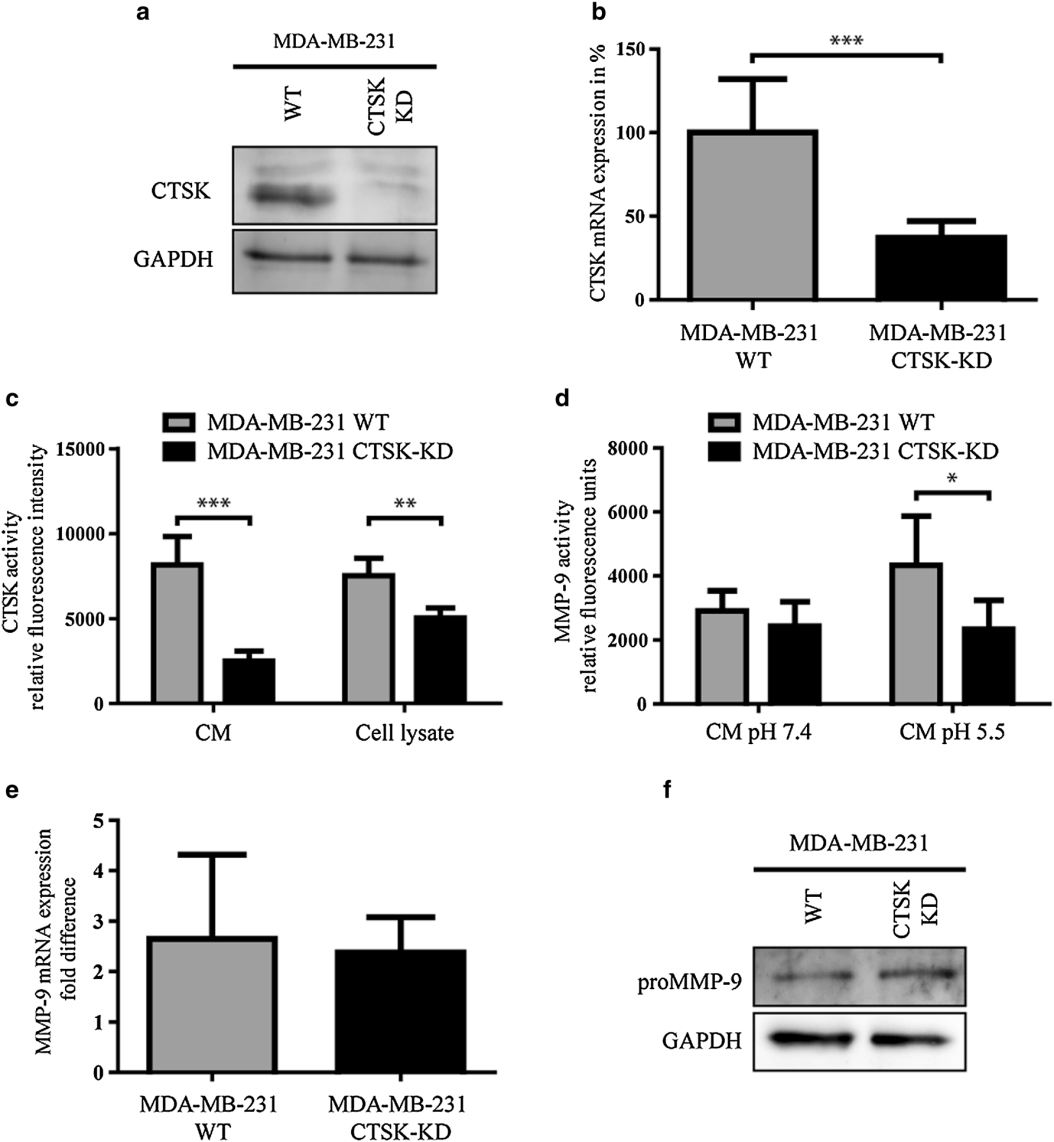
Endogenous CTSK can active endogenous MMP-9 in triple negative metastatic breast cancer cells MDA-MB-231. **a** Immunoblot of CTSK from total cell lysates of MDA-MB-231 cells transduced with scrambled mirRNA sequence (WT) or a mirRNA sequence targeting CTSK (CTSK KD). GAPDH band was used as the loading control. **b** qRT-PCR of CTSK mRNA levels in MDA-MB-231 WT and MDA-MB-231 CTSK-KD. CTSK mRNA levels were normalized to RRS18 N and the data is represented as the mean ± SD, n = 3. **c** CTSK activity was measured in conditioned media (pH 5.0) and total cell lysate (pH 5.0) from MDA-MB-231 WT and MDA-MB-231 CTSK-KD. Data is represented as the mean ± SD, n = 5. **d** MMP-9 activity as measured in the conditioned media from MDA-MB-231 WT and MDA-MB-231 CTSK-KD. Media was acidified to pH 5.5 or pH 7.4 and incubated for 1 h at 37°C, afterwards the pH was adjusted to 7.4 and MMP-9 activity was measured using a fluorescently quenched substrate for MMP-9 (Mca-RPPGFSAFK(Dnp)). Data is represented as the mean ± SD, n = 5. **e** qRT-PCR of MMP-9 mRNA levels in MDA-MB-231 WT and MDA-MB-231 CTSK-KD. MMP-9 mRNA levels were normalized to RRS18 N and data is represented as mean ± SD, n = 3. **f** Immunoblot of proMMP-9 from total cell lysates of MDA-MB-231 WT and MDA-MB-231 CTSK-KD. GAPDH band was used as loading control.*P < 0.05, **P < 0.01 and ***P < 0.005.

MMP-9 expression is thought to be a key in promoting pro-tumorigenic and pro-metastatic signaling cascades within the tumor environment [36]. Overexpression of MMP-9 is often found within tumors, where it can contribute to tumor progression through angiogenesis and increased migration of tumor cells [1, 2]. So it is possible, that CTSK within tumor environments and in bone metastatic lesions has an additional role of activating MMP-9. To test whether endogenous expression of CTSK could indeed activate tumor derived MMP-9, we examined MMP-9 activity following knockdown of CTSK mRNA using shRNA in the metastatic breast cancer cell line MDA-MB-231. The knockdown resulted in a significant reduction of CTSK mRNA expression level, which was accompanied by reduction in both cytosolic and secreted CTSK activity (Fig. 3a–c). In this scenario, lowering the pH of CM collected from MDA-MB-231 WT and MDA-MB-231 CTSK-KD yielded a significantly lower activity of MMP-9 in the CM collected from the cells depleted of CTSK (Fig. 3d). However, knockdown of CTSK did not alter MMP-9 mRNA and proteins levels (Fig. 3e, f), thus demonstrating that MDA-MB-231 secretes CTSK that it is capable of activating endogenously secreted proMMP-9. This is a significant finding, which if demonstrated in vivo could imply that cancer cells could modulate their microenvironment through CTSK-MMP-9 interplay. However, whether CTSK activation of proMMP-9 can regulate tumor progression needs to be extensively investigated in appropriate animal models before drawing broad and general conclusions.

## Conclusions

The results of this study provide the first evidence that CTSK can cleave proMMP-9 under acidic conditions. This finding is of significance as it is well established that MMP-9 promotes angiogenesis and cell migration [1, 2]. Since the tumor environment is inherently acidic due to high rate of glycolysis (Warburg Effect) the necessary trigger for secreted CTSK to act on latent proMMP-9 is ever present. Our finding that proMMP-9 and CTSK are secreted by both OCs and the metastatic breast cancer cell line MDA-MB-231 allude to a potential paradigm where the activation of proMMP-9 can occur through crosstalk between OCs resorptive pits and arriving breast cancer cells. The findings of this study provide further credence to this strategy of targeting CTSK activity to limit bone metastasis and also provide a framework for exploring more specific inhibitors of CTSK that interference with biding and activation of pro-MMP-9. However, the ability CTSK-proMMP-9 nexus to influence and alter tumor behavior needs to be verified in vivo.

## Methods

### Cell lines

The epithelial cell line MDA-MB-231 and HEK293 was cultured in Dulbecco’s modified Eagle essential medium (DMEM) Glutamax containing 10% FCS and cultured at 37°C and 5% CO_2_. MDA-MB-231 and HEK293 was provided by the Centre for Biological Signalling Studies (BIOSS) and were genotyped and verified at Labor für DNA Analytik (Freiburg, Germany).

### Lentiviral production and transduction

Lentiviral particles containing shRNA constructs (shCTSK V2LHS-92790-C5 and control RHS4346, Fisher Scientific, Germany) were produced in HEK293 cells, by co-transfecting lentiviral vector and packaging vectors using polyethylenimine (Mw 25.000, Sigma, Germany) as the transfection reagent. For transfection, 30 µg of DNA (4:3:1 of transfer vector, packaging coding vector (pCMV-dR8.74) and envelope coding vector (pMD2.G)) was diluted in 250 µL Opti-MEM (Invitrogen, Germany) and 11.25 µL of polyethylenimine (1 mg/mL) was added to the solution and the resulting mixture was incubated for 1 h at room temperature prior to adding to HEK293 cells. The medium was changed after 16 h to DMEM with 10% FCS and 40 and 64 h after transfection the viral supernatants were collected and filtered through a sterile 0.45 µm syringe filter (Millipore, Germany). Viral particles were added to target cells (MDA-MB-231) supplemented with 5 µg/mL polybrene (Sigma, Germany). 3 days after transduction, infected cells were selected by adding 5 µg/mL puromycin (Sigma, Germany) to the culture medium.

### Monocyte isolation and differentiation

Buffy coats were procured from donated blood as per the guidelines of the University Clinic at the University Freiburg. PMBC were isolated from buffy coats by Ficoll-Paque according to manufactures protocol (VWR, Germany), and CD14^+^ monocytes were isolated using CD14 magnetically labeled micro beads as per the manufactures protocol (Miltenyi Biotec, Germany). Monocytes were seeded at a concentration of 1.5 × 10^6^ cell/cm^2^ and cultured for 4 days in alpha-MEM (Invitrogen, Germany) basic medium (HEPES, sodium pyruvate, penicillin–streptomycin-glutamine, 10% FCS) supplemented with 25 ng/mL rhM-CSF (RnD Systems, Germany) after which the media was supplemented with 50 ng/mL rhRANK-L (RnD Systems, Germany). Media was changed every 4 days, osteoclast formation was observed after 4 weeks of culture. Osteoclasts were stained with tartrate-resistant acid phosphatase (TRAP) staining kit (Sigma, Germany) according to manufactures protocol. The nuclei were stained with DAPI.

### Quantitative real-time PCR

Total RNA was extracted using RNeasy Mini Kit (Qiagen). Reverse transcription of mRNA to cDNA was done using QuantiTect reverse transcription kit (Qiagen). Quantitative real-time PCR was performed using Rotor-Gene SYBR-Green PCR assay (Qiagen, Germany) on a Rotor-Gene Q machine (Qiagen, Germany), with the following thermal cycling conditions: 2 min at 50°C, 10 min at 95°C, followed by 40 cycles at 95°C for 15 s and 60°C for 1 min. All biological samples were measured in triplicates, and the resulting data were normalized to RRS18 N. The following qRT-PCR primers were used ACP5_F: AATGTCTCTGCCCAGATTGC, ACP5_R: GAAGTGCAGGCGGTAGAAAG, CTSK_F: CCGCAGTAATGACACCCTTT, CTSK_R: GCACCCACAGAGCTAAAAGC, MMP-9_F: CATCGTCATCCAGTTTGGTG, MMP-9_R: AGGGACCACAACTCGTCATC, RRS18N_F: CGGCTACCACATCCAAGGAA and RRS18N_R: GCTGGAATTACCGCGGCT.

### Western blot

For immunoblotting, cells were lysed in Laemmli buffer, and protein concentration was determined using Pierce BCA protein assay kit (Life Technologies). 20 μg of protein was loaded per sample and resolved on a 8% (wt/vol) polyacrylamide–SDS gel and transferred onto nitrocellulose membranes, which were immediately blocked with 3% (w/v) BSA for 1 h. Membranes were then probed with an antibody against CTSK (Millipore, MAB3324) or proMMP-9 (R&D systems, MAB9111). GAPDH (Santa Cruz Biotechnology, SC-25778) was used as a loading control. Blots were developed using peroxidase-conjugated secondary antibodies and chemiluminescence system (Thermo Scientific).

### Zymography

Electrophoresis was performed with 10% polyacrylamide/sodium dodecyl sulfate gels containing 1 mg/ml of gelatin (Sigma, Germany). Sodium dodecyl sulfate was removed by washing in 2.5% Triton X-100 for 1 h at room temperature before the enzyme reaction. The gel was incubated overnight at 37°C in enzyme buffer containing 50 mmol/L Tris, pH 7.5, 200 mmol/L NaCl, 5 mmol/L CaCl_2_, and 0.02% Brij-35. Area of gelatin degradation, identified as MMP activity, appeared as distinct white band after staining the gel with simply blue (Invitrogen, Germany). The intensities of both pro- and active MMP-9 were documented by a digital gel-imaging system (Thermo Scientific) and bands were analyzed by densitometry using imageJ.

### Enzyme activity

CTSK activity was measured using a CTSK activity assay kit (Biovision Research Products, Germany). The fluorescence-based assay uses a preferred CTSK substrate sequence labeled with amino-4-trifluoromethyl Coumarin. The substrate was incubated with whole tissue lysate or CM in reaction buffer at 37°C for 1 h. The specificity of the assay was confirmed by incubating samples in the presence of cathepsin-specific inhibitors (K141-100-4-BV, Biovision Research Products, Germany) [37]. The fluorescence was measured with 400-nm excitation filter and 528-nm emission filter. MMP-9 activity was measured using the fluorescence substrate Mca-RPPGFSAFK(Dnp) (Enzo Life sciences, Germany). CM or rhMMP-9 was incubated with PBS with Mg^2+^ and Ca^2+^ for 4 h and fluorescence was measured with 320-nm excitation filter and 400-nm emission filter. MMP-3 activity was measured using a MMP-3 activity assay kit (K783-100-BV, Biovision Research Products, Germany) according to manufactures instructions. The fluorescence was measured with 320-nm excitation filter and 400-nm emission filter. rhCTSK (BML-SE553-0010) and rhproMMP-9 (ALX-200-430-C010) was purchased from Enzo Life Sciences and MMP-3 was obtained from Life Technologies (10467-HNAE-5).

### Statistics

Data are shown as mean ± SD. Statistical analysis was done using GraphPad Prism software. A 2 tailed Student’s *t* test were used for all comparisons. P < 0.05 was considered significant.

## Additional file

Additional file 1: MMP-3 activity in osteoclast conditioned media. MMP-3 activity in osteoclasts conditioned media was measured using a fluorescence activity kit. Recombinant human MMP-3 at various concentrations was used as a control. The data is presented as the mean ± SD, n = 3.
