# Supplementary figures and images for: Matrix-metalloproteinase-9 is cleaved and activated by Cathepsin K
Source: BMC Res Notes. 2015 Jul 29;8:322. doi: 10.1186/s13104-015-1284-8 (PMC4518881; doi:10.1186/s13104-015-1284-8)

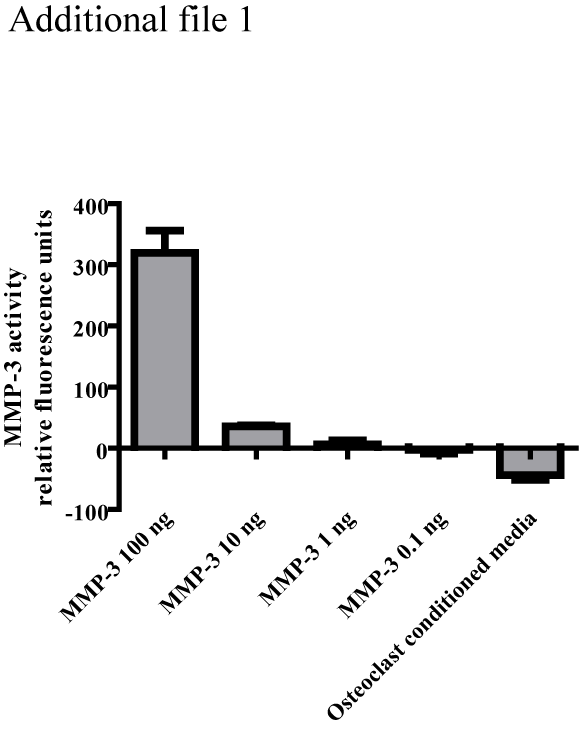

Supplement: Additional file 1: — MMP-3 activity in osteoclast conditioned media. MMP-3 activity in osteoclasts conditioned media was measured using a fluorescence activity kit. Recombinant human MMP-3 at various concentrations was used as a control. The data is presented as the mean ± SD, n = 3. [file 13104_2015_1284_MOESM1_ESM.png]
